# Myélite aigue et hépatite virale A: à propos d’un cas

**DOI:** 10.11604/pamj.2016.25.71.10265

**Published:** 2016-10-04

**Authors:** Tarik Boulahri, Ahmed Bourazza

**Affiliations:** 1 Service de Neurologie, Hôpital Militaire de Guelmim, Maroc; 2 Service de Neurologie, Hôpital Militaire d’Instruction Mohamed V, Rabat, Maroc

**Keywords:** Ataxie, myélite, hépatite A, Ataxia, myelitis, hepatitis A

## Abstract

Le virus de l’hépatite A peut se manifester par une atteinte neurologique sévère et inhabituelle. Nous rapportons l’observation d’un homme de 59 ans, qui a présenté un trouble de l’équilibre associé à des paresthésies des membres inferieures et des troubles sphinctériens. L’IRM médullaire était en faveur d’une myélite aigue. Le bilan étiologique a trouvé comme seule anomalie la présence d’IgM spécifiques du virus de l’hépatite virale A (HVA). Malgré une corticothérapie à forte dose, la récupération fonctionnelle n’était que partielle. Devant toute myélite aigue, la recherche d’une infection par le virus de l’hépatite A devrait faire partie de l’enquête étiologique même en l’absence de signe clinique ou biologique évoquant l’hépatite en particulier dans les pays d’endémie où la prophylaxie vaccinale fait défaut.

## Abstract (en)

Hepatitis A virus can manifest with severe and unusual neurological attack. We report the case of a 59-year old man with balance disorder associated with lower limb paresthesias and sphincter disorders. Spinal MRI was in favor of acute myelitis. Etiologic assessment showed viral hepatitis A (HVA) -specific IgM virus as the sole abnormality. Despite high-dose corticosteroid therapy, only partial recovery was seen. A systematic etiologic search for infection caused by hepatitis A virus (HAV) should be performed in every patient with acute myelitis even in the absence of clinical or biological signs suggestive of hepatitis, especially in endemic countries where vaccine prophylaxis is lacking.

## Introduction

L’infection par le virus de l’hépatite A (HVA) est très fréquente et généralement bénigne. Elle est asymptomatique neuf fois sur dix. Rarement, dans 0,1% des cas, elle réalise une hépatite fulminante parfois fatale. Exceptionnellement elle est responsable des tableaux inhabituels avec des manifestations extra hépatiques dont certaines neurologique comme le cas de myélite aigue. Nous rapportons le cas d’un patient présentant un tableau de myélite aigue ayant seulement comme anormal dans son bilan une positivité de la sérologie de l’hépatite virale A.

## Patient et observation

**Figure 1:**
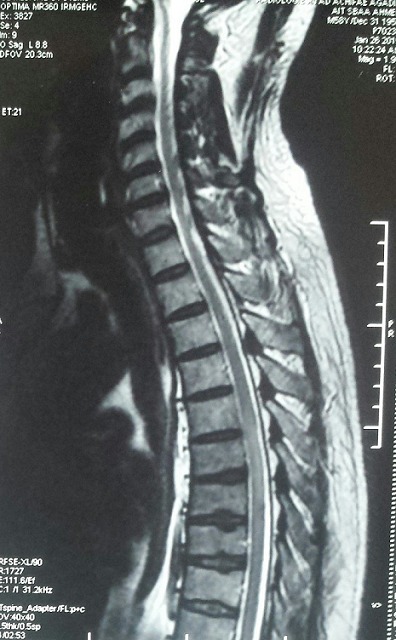
IRM médullaire séquence pondéré T2 coupe sagittale: hyper signal focal étendu sur une hauteur de 40mm de D5 à D7

**Figure 2:**
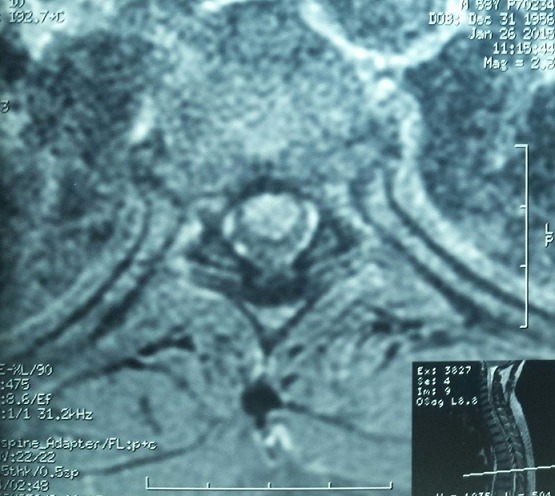
IRM médullaire en coupe axiale pondérée T2, hypersignal dorsal des cordons postérieurs

Un homme âgé de 59 ans a été admis au service de neurologie pour un trouble de l’équilibre associé à des paresthésies des membres inferieurs et des troubles sphinctériens à type d’impériosité mictionnelle et une constipation évoluant depuis 15 jours dans un contexte d’apyrexie. L’interrogatoire n’a retrouvé aucun antécédent pathologique en particulier la notion d’éruption cutanée, de troubles du transit, d’ictère, d’épisode infectieux respiratoire ou de vaccination récente. L’examen clinique à l’admission a trouvé un patient en bon état général, apyrétique à 37°C. Les constantes hémodynamiques et respiratoires étaient normales. L’examen neurologique a trouvé un patient conscient, nuque souple présentantune ataxie proprioceptive. Les réflexes ostéotendineux rotuliens et achilléens étaient abolies. La sensibilité superficielle et profonde était diminuée aux membres inferieurs avec un niveau sensitif à D 6. La force musculaire aux quatre membres était conservée. Il n’y avait pas de trouble de la coordination ni signes d’atteinte des nerfs crâniens ni atteinte des fonctions supérieures ni syndrome rachidien en regard de D6. L’IRM médullaire a montré une anomalie de signal focale intra médullaire étendu de D5 à D7 intéressant les cordons médullaires postérieurs en faveur d’une myélite aiguë (Figure 1, Figure 2). L’IRM cérébrale était normale. L’étude du liquide céphalorachidien a objectivé: 40 éléments/mm^3^ dont 80% de lymphocytes, une albuminorachie à 0,5 g/l, une glycorachie à 0,63 g/l, l’examen direct et la culture sur milieux usuels étaient négatifs, la recherche virale et du bacille de Koch par PCR dans le LCR était négative et l’électrophorèse des protides du LCR non faite. La numération formule sanguineet la vitesse de sédimentation étaient normales. Le bilan hépatique a montré un taux de bilirubine totale à 5,081m/l, Bilirubine direct: 2,09mg/l, un temps de prothrombine à 90%, des ASAT à 16 UI/l, des ALAT à 20 UI/l, des phosphatases alcalines à 239 UI/l, et des gammaglutamyl transférase à 9 UI/l. Dans le cadre de l’enquête étiologique: le bilan immunologique, les anticorps anti NMO et le taux de l’enzyme de conversion de l’angiotensine étaient normaux. L’étude anatomopathologique de la biopsie de la glande salivaire accessoire n’a pas montré d’anomalie histologique. La radiographie pulmonaire de face et la tomodensitométrie pulmonaire étaient normales. L’examen ophtalmologique ainsi que les PEV étaient normaux; le bilan thyroïdien le taux de la vitamine B12 et des folates étaient normaux; la sérologie HIV et de la syphilis était négative ; la recherche des anticorps de type immunoglobulines M dirigés contre *Mycoplasmapneumoniae, la Borreliaburgdoferi, Rickettsiaconorii,* le virus d’Epstein-Barr, l’herpes virus (1 et 2), le cytomégalovirus, le virus zona varicelle et de l’hépatite C et B était négative. Alors que la sérologie de l’hépatite A a mis en évidence la présence des anticorps anti-HVA de type immunoglobulines M. Le diagnostic retenu était donc celui d’une myélite aiguë survenant au décours d’une hépatite aiguë A anectérique. Lepatient a été mis sous corticothérapie à fortes doses (bolus de méthyl prednisolone à la dose de 1 g par jour pendant cinq jours) associée à une rééducation motrice et un traitement symptomatique de la douleur. L’évolution a été marquée par la récupération partielle de trouble de l’équilibre et des troubles sphinctériens et des paresthésies.

## Discussion

L’hépatite virale A est une affection bénigne très fréquente, survenant essentiellement dans l’enfance et souvent asymptomatique. Lorsqu’elle est symptomatique, elle se manifeste par un syndrome pseudo grippal, un syndrome dyspeptique et un ictère. Cependant, des hépatites fulminantes ont été décrites. Exceptionnellement, elle peut se compliquer d’une atteinte neurologique centrale ou périphérique. Parmi les atteintes nerveuses périphériques, la complication la plus fréquemment rapportée est le syndrome de Guillain-Barre [1, 2]. Johnston et al. ont rapporté une série de huit cas. La sérologie HVA était positive dans le sang chez tous les patients pour les IgM et dans le LCR des deux cas ou elle fut réalisée. Une observation de neuropathie motrice axonale aigue a été rapportée par Hubber et al [3]. L’atteinte nerveuse centrale est plus rare. A notre connaissance, quatre cas d’EMAD ont été rapportés dans la littérature (Johnston et al 1981; Hubber et al. 1999; Hu Seyin et al. [1, 3, 4]. Il faut également citer une observation de syndrome de Devic associé à une hépatite virale A [5]. Concernant la myélite aigue, quatre cas ont été rapporté dans la littérature, Le premier cas de myélite secondaire à une hépatite virale A confirmée par une sérologie, a été rapporté chez un adulte, en 1986 [6]. Deux autres cas de myélite aiguë associés à une hépatite virale A ont été rapportés en 1995, dont une survenant chez un enfant de quatre ans [7] et l’autre chez un adulte [8]. Le dernier cas a été rapporté chez une fille de 11 ans en 2007 [9]. Contrairement à la plupart des cas publiés, chez notre patient le tableau de myélite aigue n’était pas précédé ou contemporain à un ictère ou troubles digestifs. L’IRM médullaire montre classiquement des hyposignaux en T1 et des hypersignaux en T2. La localisation centromédullaire des anomalies du signal serait en faveur de l’origine infectieuse, alors que les localisations postérieures et/ou latérales orienteraient vers une sclérose en plaques [10]. Toutefois, la spécificité de cette topographie postérieure et/ou latérale pour le diagnostic de SEP n’est pas bonne car nous l’avons observée chez notre patient. L’étude du LCR montre une réaction lymphocytaire dans la majorité des cas, avec une glycorachie normale et une protéinorachie normale ou modérément augmentée. Chez notre patient, devant la négativité du bilan de premier intention visant à rechercher les maladies inflammatoires systémiques et celles du système nerveux central et les maladies métaboliques, le diagnostic d’hépatite aiguë A a été posé lors d’une enquête virale faite de façon systématique malgré l’absence de signes cliniques ou biologiques évoquant l’hépatite. Actuellement, trois hypothèses sont proposées pour expliquer la pathogénie de la myélite transverse: une réponse auto-immune post infectieuse, une invasion directe par le virus et une occlusion vasculaire aiguë [11, 12]. Un mécanisme auto-immun a été rapporté sur des données de la biopsie de la moelle épinière, dans un cas de myélite aiguë secondaire à une hépatite virale C [13]. Dans notre cas, on retient a priori un mécanisme auto-immun post viral, vu l’absence de signes cliniques ou biologiques en faveur d’une hépatite aiguë évolutive au moment du diagnostic de la myélite.

## Conclusion

L’association myélite aigue et hépatite A est exceptionnelle. Toutefois, la recherche d’une infection par le virus de l’hépatite A devrait faire partie de l’enquête étiologique d’une myélite aiguë même en l’absence de signe clinique ou biologique évoquant l’hépatite en particulier dans les pays d’endémie et où la prophylaxie vaccinale fait défaut.
